# Fears and desires: a quantitative analysis of psychological, relational, and sociocultural factors in women’s desire to have children

**DOI:** 10.1186/s12889-026-27476-0

**Published:** 2026-04-20

**Authors:** Aylin Arıcı

**Affiliations:** 1 Social Work Department, Istanbul University-Cerrahpasa, Istanbul, Turkey; 2 UNEC Social Work and Social Innovations Research Center, Azerbaijan State University of Economics, Baku, Azerbaijan

**Keywords:** Desire to have children, Women, Marital satisfaction, Emotions, Social support, Gender role attitudes

## Abstract

**Objective:**

This study aimed to examine the relationships between women’s desire to have children and their marital satisfaction, emotional state (positive and negative affect), perceived social support, and gender role attitudes.

**Method:**

The research was conducted with 1,582 married women across seven geographical regions of Türkiye. Data were collected through an online questionnaire using a multi-center snowball sampling method. Valid and reliable scales were used (α = 0.78–0.92), and correlation and regression analyses were performed.

**Results:**

The desire to have children was negatively correlated with marital satisfaction (*r* = –.112, *p* < .01) and positive affect (*r* = –.065, *p* < .01), but positively correlated with negative affect (*r* = .141, *p* < .01) and gender role attitudes (*r* = .432, *p* < .01). Perceived social support was not statistically significant (*r* = –.016, *p* > .05). Regression analysis revealed that egalitarian gender role attitudes were the strongest predictor, explaining 18.7% of the variance.

**Conclusion:**

The desire to have children cannot be explained solely by marital satisfaction or psychological well-being. Instead, it is shaped by complex mechanisms such as emotional regulation, relational needs, identity formation, and gender norms. The enhancing role of negative affect and the diminishing effect of positive affect suggest that fertility motivations go beyond conventional well-being models. Moreover, the predictive strength of egalitarian gender attitudes underscores the importance of social norms and gender equality. These findings highlight the need for gender-sensitive policies that center women’s subjective experiences in addressing fertility decline.

## Introduction

The decision to have children is not only one of the most significant milestones in an individual’s life course but also a key determinant of the demographic and social sustainability of societies. In the 21st century, the decline of fertility rates below the population replacement level in many countries has made it essential to understand the psychological, relational, and cultural dynamics underlying this decision [1]. According to OECD data, the average total fertility rate across member countries fell to 1.43 in 2023, leading to serious global consequences such as population aging, a shrinking labor force, and an increased burden of care [2]. Similar trends observed across Europe, Asia, and the Americas suggest that low fertility is not solely driven by economic factors but is closely related to individuals’ subjective well-being, the quality of marital relationships, gender norms, and social support networks.

Türkiye is also part of this global trend. According to the Turkish Statistical Institute [3], the total fertility rate declined from 2.38 in 2001 to 1.48 in 2024. Moreover, the age at first birth has increased, and childbearing has been increasingly postponed. In Türkiye—where family and motherhood have historically been highly valued—this decline has not only demographic but also notable psychosocial implications [4]. In this context, understanding women’s desire to have children holds critical importance, not only for Türkiye but also for addressing the global fertility crisis. Women, beyond being biological actors in reproductive processes, are decision-makers who engage with relational satisfaction, emotional experience, social support mechanisms, and gender norms. Therefore, studies focusing on women’s desire to have children make an original contribution at both theoretical and practical levels by illuminating the tension between individual subjectivity and structural conditions.

In this study, women’s desire to have children is examined through four key psychosocial variables: marital satisfaction, affective state (positive and negative emotions), perceived social support, and gender role attitudes. Although each of these variables has been explored independently in the literature, their joint examination within a holistic model remains limited. Marital satisfaction is strongly associated with the desire to have children. The meta-analysis by Twenge, Campbell, and Foster revealed that the transition to parenthood often decreases marital satisfaction, while other studies suggest that in marriages with lower satisfaction levels, the desire for children may serve a compensatory relational function [5, 6].

Affective state is another psychological factor shaping parenting motivations. Although positive emotions are proposed to increase the desire for children [7], negative emotions have also been found to trigger this desire. Brase and Brase’s concept of *baby fever* draws attention to the link between the desire for children and intense emotional impulses [8]. In this regard, parenthood can be considered not only a reflection of favorable life conditions but also a means of coping with negative emotions and generating existential meaning. Perceived social support is often viewed as a facilitating factor in decisions about parenthood; findings vary across contexts. Studies conducted in Europe and Iran indicate that social support becomes more salient in decisions about having a second or third child, while demographic factors often exert stronger influence [9, 10]. Graham et al. further demonstrated that women mobilize support not from a single source but from multiple people, for different purposes, and at different stages of the reproductive process [11]. Gender role attitudes represent one of the most powerful normative factors shaping women’s desire to have children. While evidence from Europe suggests that egalitarian attitudes are associated with higher fertility [12, 13], traditional values have been found to play a stronger role in contexts such as China and Iran [14, 15]. The study by Arpino, Esping-Andersen, and Pessin further demonstrated that the relationship between gender equality and fertility follows a non-linear, U-shaped pattern. This finding underscores that the social norms influencing women’s reproductive desires are highly context-sensitive and dynamic [16].

From a conceptual perspective, women’s desire to have children can be understood as a multidimensional construct shaped by the interaction of psychological, relational, and sociocultural domains. The present study is positioned within ecological and contextual perspectives, which conceptualize human behavior as emerging from the dynamic interplay of multiple levels of influence. At the individual level, emotional states and subjective well-being (e.g., positive and negative affect) shape personal motivations and expectations. At the relational level, marital satisfaction and perceived social support influence how individuals evaluate and experience family-related decisions. At the broader societal level, gender role attitudes and prevailing cultural norms structure expectations regarding motherhood and reproductive behavior. These levels do not operate independently; rather, they interact to shape women’s reproductive decision-making processes.

Although prior research has examined the desire to have children through various individual or contextual variables, these factors have predominantly been studied in isolation, with limited attention to their interconnected and cross-level nature. However, marital satisfaction, affective state, perceived social support, and gender role attitudes represent distinct yet interrelated dimensions of fertility decision-making. Relational quality and perceived support influence individuals’ evaluation of family life and available resources, while affective processes provide the emotional and motivational basis underlying these evaluations. At the same time, gender role attitudes constitute the broader normative framework within which both individual and relational processes acquire meaning. Considering these variables jointly therefore allows for a more comprehensive understanding of fertility intentions as a product of interacting psychological, relational, and sociocultural mechanisms.

In this regard, the present study addresses a gap in the literature by examining these factors together as interrelated components operating across multiple psychosocial levels. While these variables are conceptually integrated within a multidimensional framework, their predictive roles were examined individually in the analyses (i.e., not within a single integrative model) using large-scale data collected from 1,582 married women across Türkiye’s seven geographical regions. In doing so, the study contributes to both national and international literature by providing a more nuanced and comprehensive account of women’s desire to have children. Furthermore, by centering women’s lived experiences, the study sheds light on the psychosocial processes underlying declining fertility rates, offering insights relevant not only to the Turkish context but also to broader global discussions.

## The present study

Drawing upon the reviewed literature, the current study aims to examine the psychological, relational, and sociocultural determinants of married women’s desire to have children. Given the complex and context-dependent nature of fertility intentions, the hypotheses were formulated as non-directional. Based on these overarching objectives, the following hypotheses were developed and tested:

### H1

Marital satisfaction is significantly associated with the desire to have children.

### H2a

Negative affect is significantly associated with the desire to have children.

### H2b

Positive affect is significantly associated with the desire to have children.

### H3

Perceived social support is significantly associated with the desire to have children.

### H4

Gender role attitudes are significantly associated with the desire to have children.

### Method

#### Research design and research question

This study examined the psychological, social, and societal factors influencing married women’s desire to have children. The decision to have a child is not merely an individual choice; it is a multidimensional process shaped by a range of dynamics—from the quality of spousal relationships and emotional experiences to social support networks and gender role norms. Therefore, the study addressed variables at multiple levels: at the individual level (emotional state), interpersonal level (marital satisfaction and perceived social support), and sociocultural level (gender role attitudes).

The research employed a quantitative correlational design aimed at identifying the direction and magnitude of relationships among these variables [17, 18]. Within this framework, the central research question was formulated as follows:

“How do marital satisfaction, emotional state (positive and negative affect), perceived social support, and gender role attitudes influence women’s desire to have children?”

### Population and sample

The target population of the study consisted of married women residing in Türkiye. Participants were recruited using a multi-centered snowball sampling method simultaneously initiated across the country’s seven geographical regions [19, 20]. This approach was preferred to facilitate access to a geographically diverse sample and to enable recruitment through trust-based networks, particularly given the sensitive nature of the topic. At least one initial participant was reached in each region, and these individuals were asked to recommend additional participants who met the study criteria (female, aged 18 years or older, and married). Accordingly, the inclusion criteria of the study were clearly defined as being female, being married, and being 18 years of age or older. Sociodemographic variables such as income level, duration of marriage, and parental characteristics were not used as exclusion criteria; instead, their potential associations with women’s desire to have children were examined within the scope of the analyses.

When participation from certain regions remained limited during the process, new initial participants were selected from those regions to strengthen the recruitment chains. Through this approach, a total of 1582 participants were reached, representing all seven geographical regions of Türkiye (Marmara, Aegean, Mediterranean, Central Anatolia, Black Sea, Eastern Anatolia, and Southeastern Anatolia). This sampling strategy enabled the inclusion of participants from diverse sociocultural contexts across regions.

### Measures

The data collection instruments used in this study were as follows:

#### Sociodemographic information form

Developed by the researcher, this form collected basic demographic information such as participants’ age, education level, employment status, income level, duration of marriage, and number of children.

#### Desire to have children scale

Originally developed by Naghibi et al. and adapted into Turkish by Kamiloğlu and Vural, this 19-item scale employs a five-point Likert format and consists of four subdimensions [21, 22]: *Positive Motivations for Having Children*,* Preference for Having Children*,* Concerns About Having Children*, and *Social Beliefs.* In the present study, the Cronbach’s alpha coefficient was 0.82.

#### Marital life satisfaction scale

Developed by Norton and adapted into Turkish by Çelik, this unidimensional instrument includes five items rated on a five-point Likert scale. The Cronbach’s alpha coefficient in this study was 0.85 [23, 24].

#### Positive and negative affect schedule (PANAS)

Developed by Watson, Clark, and Tellegen and adapted into Turkish by Gençöz, the PANAS consists of 20 items rated on a five-point Likert scale [25, 26]. The *Positive Affect* subscale (10 items) assesses enthusiasm, energy, and interest, while the *Negative Affect* subscale (10 items) measures stress, guilt, and anxiety. In the present study, Cronbach’s alpha coefficients were 0.83 for positive affect and 0.86 for negative affect.

#### Multidimensional scale of perceived social support (MSPSS)

Developed by Zimet et al. and adapted into Turkish by Eker, Arkar, and Yaldız, this 12-item scale is rated on a seven-point Likert scale and comprises three subdimensions [27, 28] : *Family*,* Friends*, and *Significant Other.* The Cronbach’s alpha coefficient for this study was 0.89.

#### Gender roles attitude scale

Developed by Zeyneloğlu and Terzioğlu, this 38-item scale employs a five-point Likert format and covers five subdimensions [29] : *Egalitarian Gender Role*,* Female Gender Role*,* Marital Gender Role*,* Traditional Gender Role*, and *Male Gender Role.* The Cronbach’s alpha coefficient obtained in this study was 0.92.

## Data collection procedure

Data were collected between February 22, 2025, and June 12, 2025, through an online survey administered via Google Forms. The survey link was distributed through email, social media groups, and local networks. At the beginning of the survey, participants were informed about the study’s purpose, voluntary participation principles, confidentiality, and their right to withdraw at any time. Only those who provided informed consent were included in the study. Prior to data collection, a pilot study was conducted with 42 participants to ensure clarity and optimize the administration time of the questionnaire.

### Data analysis

The data were analyzed using the Statistical Package for the Social Sciences (SPSS) version 25.0. Prior to conducting the analyses, the distributional properties of the variables were examined, and the assumption of normality was tested. Based on George and Mallery’s ± 2 criterion, the data were found to meet the assumption of normal distribution [30].

First, frequency and percentage distributions regarding participants’ demographic and familial characteristics were calculated (Table 1). The internal consistency of the scales used in the study was assessed using Cronbach’s alpha coefficients, which indicated acceptable levels of reliability (Table 2). Descriptive statistics for the scales and their subdimensions—including mean, standard deviation, minimum, and maximum values—were reported (Table 3). Furthermore, independent samples t-tests and one-way analysis of variance (ANOVA) were conducted to examine the differences in the desire to have children scores across participants’ sociodemographic characteristics. The Games-Howell post-hoc test was utilized to determine the source of significant differences in the ANOVA results. To evaluate the practical significance of these differences, effect sizes were calculated using Cohen’s d for the t-tests and Eta-squared (η²) for the ANOVAs (Table 4).

In line with the main objective of the research, Pearson correlation analysis was performed to examine the relationships between the *Desire to Have Children* and other psychosocial variables. To evaluate the practical significance and strength of these findings, the magnitudes of the effect sizes (Cohen’s d and η²) and correlation coefficients (r) were interpreted based on Cohen’s established criteria (e.g., *r* = .10–0.29 as weak, *r* = .30–0.49 as moderate, and *r* ≥ .50 as strong) (Table 5) [31]. In the analyses, Positive and Negative Affect were evaluated as two independent dimensions as necessitated by the scale’s original psychometric design, whereas total scores were utilized for the remaining instruments to assess their holistic macro-constructs.

Following the correlation analyses, simple linear regression analyses were conducted to identify the factors that significantly predicted women’s desire to have children. In these analyses, marital satisfaction (Table 6), negative affect (Table 7), positive affect (Table 8), and gender role attitudes (Table 9) were independently evaluated as independent variables in separate regression models. For each regression model, model significance (F-test), explanatory power (R² and Adjusted R²), and the predictive strength of the independent variable (β coefficient) were evaluated. A significance level of *p* < .05 was adopted for all analyses.

## Results

This section first presents the descriptive findings regarding participants’ demographic and familial characteristics, followed by statistical analyses examining the relationships between the *Desire to Have Children* and *Marital Life Satisfaction*, *Positive and Negative Affect*, *Perceived Social Support*, and *Gender Role Attitudes*.

### Findings on participants’ sociodemographic and family characteristics

Table 1 presents the demographic characteristics of the participants. The mean age of the participants was 35.43 years (SD = 7.83). Categorically, the largest group (38.4%) was between the ages of 30 and 39. In terms of education, the largest group consisted of Bachelor’s degree holders (41.5%), followed by high school graduates (27.5%). Regarding income levels, the highest proportion (40.6%) fell within the 30,000–59,999 TL range, while the lowest proportion (1.1%) was in the 0–9,999 TL range. More than half of the participants (59.4%) reported not being employed. A majority of participants (70.2%) resided in the Marmara Region, and 89.8% lived in urban centers. Regarding the duration of marriage, the highest proportion (34.8%) had been married for 16 years or more, followed by 23.2% who had been married for 1–5 years. Most participants had children (79.5%), while 20.5% did not. Among mothers, 31.7% had two children, 26.2% had one child, and 21.6% had three or more children. Additionally, most participants (85.8%) reported no chronic illness, and 50.6% indicated using a birth control method.

**Table 1:** Distribution of Participants by Marital Duration, Parental Status, and Other Sociodemographic Characteristics

Characteristics	*N*	%
Age*		
18–29 years	433	27.4
30–39 years	608	38.4
40–49 years	541	34.2
**Education Level**		
Primary school	252	15.9
Secondary school	167	10.6
High school	435	27.5
Bachelor’s Degree	656	41.5
Postgraduate	72	4.6
**Income Level**		
No income/Unspecified	29	1.8
0-9.999 TRY	18	1.1
10.000-29.999 TRY	223	14.1
30.000-59.999 TRY	642	40.6
60.000-99.999 TRY	422	26.7
100.000 TRY and above	248	15.7
**Employment Status**		
Employed	643	40.6
Unemployed	939	59.4
**Place of residence**		
Rural area	161	10.2
Urban center	1421	89.8
**Region of residence**		
Marmara Region	1119	70.7
Aegean Region	82	5.2
Mediterranean Region	67	4.2
Central Anatolia Region	114	7.2
Black Sea Region	96	6.1
Eastern Anatolia Region	84	5.3
Southeastern Anatolia Region	20	1.3
**Duration of Marriage**		
Less than 1 year	147	9.3
1–5 years	367	23.2
6–10 years	260	16.4
11–15 years	258	16.3
16 years and more	550	34.8
**Parental status**		
Yes	1257	79.5
No	325	20.5
**Number of children**		
No	325	20.5
1 child	415	26.2
2 children	501	31.7
3 or more children	341	21.6
**Chronic Illness**		
Yes	225	14.2
No	1357	85.8
**Use of contraceptive methods**		
Yes	782	49.4
No	800	50.6
**Total**	1582	100

Note. TRY: Turkish Lira. *Age groups were established based on reproductive life stages (20s as traditional, 30s as postponement, and 40 + as late fertility) as conceptualized in current demographic literature [32]

### Reliability analysis and descriptive statistics of the scales

Table 2 presents the internal consistency reliability coefficients of the scales used in the study. The overall Cronbach’s alpha coefficient was calculated as 0.700 for the *Desire to Have Children Scale*, 0.781 for the *Marital Life Satisfaction Scale*, 0.914 for the *Multidimensional Scale of Perceived Social Support*, and 0.917 for the *Gender Roles Attitude Scale*. For the *Positive and Negative Affect Scale*, which consists of two independent subscales, the reliability coefficients were 0.841 for *Positive Affect* and 0.847 for *Negative Affect*.

**Table 2:** Results of Reliability Analysis for the Scales

Scale		Number of Items	Cronbach’s α
Desire to Have Children Scale (Total)		19	0.700
Marital Life Satisfaction Scale (Total)		5	0.781
Positive and Negative Affect Scale		20	
	Positive Affect	10	0.841
	Negative Affect	10	0.847
Multidimensional Perceived Social Support Scale		12	0.914
Gender Roles Attitude Scale		38	0.917

Table 3 presents the number of items, mean (M), standard deviation (SD), minimum (Min.), and maximum (Max.) values for the scales and their subdimensions used in the study. The mean total score for the *Desire to Have Children Scale* was 55.74, with the highest subscale mean observed for *Positive Motivations for Having Children* (M = 20.23) and the lowest for *Preference for Having Children* (M = 7.29). The *Marital Life Satisfaction Scale* had a mean score of 22.95. For the *Positive and Negative Affect Scale*, *Positive Affect* (M = 32.02) showed a higher mean than *Negative Affect* (M = 20.60). The mean total score for the *Multidimensional Scale of Perceived Social Support* was 60.49, with the highest subscale mean belonging to *Significant Other* (M = 20.72) and the lowest to *Family* (M = 19.78). Finally, the *Gender Roles Attitude Scale* had a total mean score of 149.73, with the highest mean observed for the *Marital Gender Role* subscale (M = 35.66).

**Table 3:** Descriptive statistics for the scales and their subdimensions

Scales	Subscales	No. of Items	Mean (M)	SD	Min	Max
Desire to Have Children Scale	Positive Motivations for Having Children	7	20.23	4.98	7.00	35.00
	Preference for Having Children	3	7.29	2.21	3.00	15.00
	Concerns about Having Children	4	11.69	3.40	4.00	20.00
	Social Beliefs	5	16.52	2.96	8.00	25.00
	Total Score	19	55.74	8.32	23.00	87.00
Marital Life Satisfaction Scale	Marital Life Satisfaction	5	22.95	5.94	5.00	35.00
Positive and Negative Affect Scale	Positive Affect	10	32.02	7.41	10.00	50.00
	Negative Affect	10	20.60	7.33	10.00	50.00
Multidimensional Perceived Social Support Scale	Family	4	19.78	4.56	4.00	28.00
	Friends	4	20.00	4.44	4.00	28.00
	Significant Other	4	20.72	4.30	4.00	28.00
	Total Score	12	60.49	12.17	12.00	84.00
Gender Roles Attitude Scale	Egalitarian Gender Role	8	34.76	5.10	8.00	40.00
	Female Gender Role	8	27.66	6.07	8.00	40.00
	Gender Role in Marriage	8	35.66	4.34	8.00	40.00
	Traditional Gender Role	8	27.64	6.15	8.00	40.00
	Male Gender Role	6	24.02	3.93	6.00	30.00
	Total Score	38	149.73	19.92	38.00	190.00

*N* = 1582

Table 4 presents the comparative analysis results of the participants’ desire to have children scores based on their sociodemographic characteristics. Regarding education level, the desire to have children scores of bachelor’s degree (*M* = 58.13) and postgraduate (*M* = 57.81) degree holders were found to be statistically significantly higher than those of primary, secondary, and high school graduates (*F*(4, 1577) = 34.412, *p* < .001). Effect size analysis indicates that education level has a medium effect (*η²* = 0.08) on the desire to have children.

**Table 4:** Comparison of Desire to Have Children Scores Across Sociodemographic Characteristics

Variables	Groups	*N*	Mean ± SD	t/F	*p*	Effect Size	Post-hoc tests
Education Level	Primary school (1)	252	52.11 ± 7.44	34.412	< 0.001	η² = 0.08	4 > 1,2,3 5 > 1,2,3 3 > 1 (Games-Howell)
	Secondary school (2)	167	53.47 ± 7.38				
	High school (3)	435	54.82 ± 7.71				
	Bachelor’s Degree (4)	656	58.13 ± 8.59				
	Postgraduate (5)	72	57.81 ± 8.35				
Parental status	Yes	1257	54.72 ± 7.81	-8.893	< 0.001	d = 0.58	-
	No	325	59.66 ± 9.27				
Employment Status	Employed	643	57.70 ± 8.51	8.02	< 0.001	d = 0.40	-
	Unemployed	939	54.38 ± 7.99				
Number of children	No (1)	325	59.66 ± 9.27	59.565	< 0.001	η² = 0.10	1 > 2>3 > 4 (Games-Howell)
	1 child (2)	415	57.20 ± 7.99				
	2 children (3)	501	54.16 ± 7.70				
	3 or more children (4)	341	52.52 ± 6.74				
Duration of Marriage	Less than 1 year (1)	147	58.62 ± 8.97	26.35	< 0.001	η² = 0.06	1,2,3,4 > 5 (Games-Howell)
	1–5 years (2)	367	57.73 ± 9.35				
	6–10 years (3)	260	56.56 ± 7.92				
	11–15 years (4)	258	56.24 ± 7.89				
	16 years and more (5)	550	53.12 ± 7.17				
Chronic Illness	Yes	225	54.18 ± 7.70	-3.110	0.002	d = 0.22	-
	No	1357	55.99 ± 8.45				
Use of contraceptive methods	Yes	782	57.36 ± 8.28	7.818	< 0.001	d = 0.39	-
	No	800	54.17 ± 8.14				

*N* = 1582. F: One-way Anova, t: Independent Samples t-test, d: Cohen’s d

η²=Eta Squared

In terms of parental status, participants without children reported a significantly higher desire to have children (*M* = 59.66) compared to those who already have children (*M* = 54.72) (*t*(1580) = -8.893, *p* < .001), representing a medium effect size (*d* = 0.58). Similarly, as the number of children increases, the desire to have children significantly and gradually decreases (*F*(3, 1578) = 59.565, *p* < .001). Participants with no children had the highest scores (*M* = 59.66), followed by those with one child (*M* = 57.20), two children (*M* = 54.16), and three or more children (M = 52.52), respectively. This finding points to a medium-to-large effect size (*η²* = 0.10).

Examining the duration of marriage, the group married for 16 years and more (*M* = 53.12) demonstrated a significantly lower desire to have children than all other groups with shorter marriage durations (*F*(4, 1577) = 26.35, *p* < .001, *η²* = 0.06). In the context of employment, employed individuals (*M* = 57.70) exhibited significantly higher scores compared to unemployed ones (*M* = 54.38) (*t*(1580) = 8.02, *p* < .001, *d* = 0.40). Furthermore, those using contraceptive methods (*M* = 57.36) were found to have a higher desire to have children than those who do not (*M* = 54.17) (*t*(1580) = 7.818, *p* < .001, *d* = 0.39). Finally, although the desire to have children among participants without a chronic illness (*M* = 55.99) was found to be significantly higher than those with a chronic illness (*M* = 54.18) (*t*(1580) = -3.110, *p* = .002), the effect size of this variable is small (*d* = 0.22).

Table 5 presents the Pearson correlation coefficients examining the relationships between the *Desire to Have Children* and *Marital Life Satisfaction*, *Positive and Negative Affect*, *Perceived Social Support*, and *Gender Role Attitudes*. According to the results, the desire to have children was negatively correlated with *Marital Life Satisfaction* (*r* = –.112, *p* < .01) and *Positive Affect* (*r* = –.065, *p* < .01), but positively correlated with *Negative Affect* (*r* = .141, *p* < .01) and *Gender Role Attitudes* (*r* = .432, *p* < .01). No significant relationship was found between *Perceived Social Support* and the desire to have children (*r* = –.016, *p* > .05).

**Table 5:** Pearson correlation results among the study variables

Variables	1	2	3	4	5	6.
1. Desire to Have Children	-	− 0.112**	− 0.065**	0.141**	− 0.016	0.432**
2. Marital Life Satisfaction	− 0.112**	-	0.245**	− 0.257**	0.358**	0.141**
3. Positive Affect	− 0.065**	0.245**	-	− 0.091**	0.270**	0.083**
4. Negative Affect	0.141**	− 0.257**	− 0.091**	-	− 0.209**	− 0.086**
5. Multidimensional Perceived Social Support	− 0.016	0.358**	0.270**	− 0.209**	-	0.245**
6. Gender Roles Attitude	0.432**	0.141**	0.083**	− 0.086**	0.245**	-

*p < .05, **p < .01

Table 6 presents the results of the regression analysis examining the predictive effect of *Marital Life Satisfaction* on the *Desire to Have Children*. The model was statistically significant (*F*(1, 1580) = 20.062, *p* < .001). *Marital Life Satisfaction* was identified as a significant predictor of the desire to have children (β = − 0.112, *p* < .001). The model explained 1.3% of the total variance (*R²* = 0.013; *Adjusted R²* = 0.012). This finding indicates that as marital satisfaction decreases, the desire to have children increases.

**Table 6:** Results of the regression analysis examining the predictive effect of marital satisfaction on the desire to have children

Model	B	Standard Error (SE)	Beta (β)	t	*p*
Constant	59.336	0.830	-	71.478	< 0.001
Marital Life Satisfaction	–0.157	0.035	–0.112	-4.479	< 0.001
	**R**	**R²**	**Adj. R²**	**F**	**p**
	0.112	0.013	0.012	20.062	< 0.001

Dependent variable: Desire to Have Children. p < .001, statistically significant

Table 7 presents the results of the regression analysis examining the predictive effect of *Negative Affect* on the *Desire to Have Children*. The model was statistically significant (*F*(1, 1580) = 32.090, *p* < .001). *Negative Affect* was identified as a significant predictor of the desire to have children (β = 0.141, *p* < .001). The model explained **2%** of the total variance (*R²* = 0.020; *Adjusted R²* = 0.019). This finding indicates that as negative affect increases, women’s desire to have children also increases.

**Table 7:** Results of the regression analysis examining the predictive effect of negative emotions on the desire to have children

Model	B	Standard Error (SE)	Beta (β)	t	*p*
Constant	52.439	0.618	-	84.885	< 0.001
Negative Emotions	0.160	0.028	0.141	5.665	< 0.001
	**R**	**R²**	**Adj. R²**	**F**	**p**
	0.141	0.020	0.019	32.090	< 0.001

Dependent variable: Desire to Have Children. p < .001, statistically significant

Table 8 presents the results of the regression analysis examining the predictive effect of *Positive Affect* on the *Desire to Have Children*. The model was statistically significant (*F*(1, 1580) = 6.656, *p* = .010). *Positive Affect* was identified as a significant predictor of the desire to have children (β = − 0.065, *p* = .010). The model explained 0.4% of the total variance (*R²* = 0.004; *Adjusted R²* = 0.004). This finding indicates that as positive affect decreases, the desire to have children increases.

**Table 8:** Results of the regression analysis examining the predictive role of positive emotions on the desire to have children

Model	B	Standard Error (SE)	Beta (β)	t	*p*
Constant	58.066	0.927	-	62.648	< 0.001
Positive Emotions	− 0.073	0.028	− 0.065	-2.580	0.010
	**R**	**R²**	**Adj. R²**	**F**	**p**
	0.065	0.004	0.004	6.656	0.010

Dependent variable: Desire to Have Children. p < .001, statistically significant

Table 9 presents the results of the regression analysis examining the predictive effect of *Gender Role Attitudes* on the *Desire to Have Children*. The model was statistically significant (*F*(1, 1580) = 362.795, *p* < .001). *Gender Role Attitudes* were identified as a significant predictor of the desire to have children (β = 0.432, *p* < .001). The model explained 18.7% of the total variance (*R²* = 0.187; *Adjusted R²* = 0.186). This finding indicates that as egalitarian gender role attitudes increase, women’s desire to have children also increases.

**Table 9:** Results of the regression analysis examining the predictive effect of gender role attitudes on the desire to have children

Model	B	Standard Error (SE)	Beta (β)	t	*p*
Constant	28.714	1.431	-	20.063	< 0.001
Gender Role Attitudes	0.180	0.009	0.432	19.047	< 0.001
	**R**	**R²**	**Adj. R²**	**F**	**p**
	0.432	0.187	0.186	362.795	< 0.001

Dependent variable: Desire to Have Children. p < .001. statistically significant

## Discussion

In this section, the findings are discussed within the framework of the relevant literature, focusing on the psychological, relational, and social dynamics shaping women’s desire to have children. The results of the present study indicated that marital satisfaction (H1), affective state (H2a and H2b), and gender role attitudes (H4) were significantly associated with women’s desire to have children, whereas perceived social support (H3) was not significantly associated. Accordingly, all hypotheses except H3 were supported. Among these findings, the relationship between marital satisfaction and the desire to have children emerges as particularly noteworthy. Specifically, the results indicate that as marital satisfaction decreases, women’s desire to have children increases. This finding suggests that women in less satisfying marriages may seek to construct relational meaning, belonging, or continuity through motherhood. Similarly, Parr’s longitudinal study based on Australian data revealed significant associations between life satisfaction and fertility; the finding that relationship satisfaction was predictive only among men indicates that women’s childbearing decisions are shaped not merely by relationship quality but also by gender role expectations, motherhood ideology, and compensatory motives [7]. Consistent with these findings, the review by Balbo, Billari, and Mills also highlighted that women, particularly those in less satisfying relationships, often view having a child as a means of seeking meaning or achieving a sense of completeness [6]. In this context, the findings of the present study suggest that children may be positioned as a compensatory source of emotional and relational fulfillment. Importantly, when considering the magnitude of the observed relationships, although several correlations were statistically significant, most effect sizes were small to moderate according to Cohen’s criteria [31]. This indicates that while these variables are meaningfully associated with women’s desire to have children, their individual contributions are relatively limited. Such findings suggest that fertility intentions are not driven by single factors but rather emerge from the interaction of multiple psychological, relational, and sociocultural processes. Therefore, even small effect sizes should not be interpreted as trivial, but rather as components of a broader, multifaceted system shaping reproductive decision-making.

Studies conducted in the Turkish context also support this interpretation. Bozkurt and Özdemir found that women’s level of idealization of motherhood was positively associated with marital adjustment and life satisfaction [33]. Thus, women may reconstruct unsatisfying marriages through motherhood, generating meaning in accordance with cultural norms and gender expectations. Frankl’s logotherapeutic perspective similarly posits that the search for meaning persists even under conditions of dissatisfaction, and that such meaning can be embodied through a loved one—for instance, a child [34]. This interpretation aligns with Villalobos and Searles’s concept of a *“compensatory connection”* [35]. Likewise, Döne demonstrated that motherhood in modern society functions as a domain for identity construction, class belonging, and social legitimacy [36]. Within this framework, the desire to have children emerges as a multilayered phenomenon that transcends individual meaning-making and becomes embedded within social and cultural systems. On the other hand, the effects of childbearing on marital adjustment remain contested. Yılmaz, İlketenci, Yılmaz, and Mamirova (2021) found that the number of children did not directly influence marital harmony; rather, problem-solving capacity and secure communication were the key determinants [37]. Meta-analytic findings by Twenge, Campbell, and Foster, as well as those of Nomaguchi and Milkie, demonstrated that parenthood often reduces marital satisfaction and increases psychological strain [5, 38]. This dynamic is particularly evident among working mothers, for whom children can simultaneously serve as a source of meaning and as a source of role conflict and stress. In summary, this finding can be interpreted in two directions: women may attempt to compensate for declining marital satisfaction through motherhood, yet the lived experience of parenthood often fails to meet such expectations and may generate new tensions. Therefore, the gap between the *desire* for motherhood and the *realities* of parenting highlights a domain that requires further examination at both individual and societal levels.

Another distinctive finding of this study is the negative association between the desire to have children and positive emotions, and its positive association with negative emotions. This result extends beyond the conventional fertility literature, which predominantly explains the desire for parenthood through life satisfaction, subjective well-being, and positive affect [7, 39]. The present findings demonstrate that childbearing decisions may be driven not only by positive emotions but also, at times, by negative emotional states.

The enhancing effect of negative emotions can be interpreted through the concepts of *compensatory connection* [40] and *baby fever* [8]. Women experiencing uncertainty in work, relationships, or social life may turn to the mother–child bond as a compensatory source of meaning, or may experience a strong desire for a child driven by intense emotional impulses. In this sense, negative affect may serve a psychologically restorative function by fostering belonging and continuity. Conversely, the decreasing effect of positive emotions on the desire to have children is also supported in the literature. Parr found that individuals with higher life satisfaction may avoid parenthood due to concerns about disrupting their current stability [7]. Similarly, the meta-analysis by Twenge et al. showed that parenthood often decreases marital satisfaction and contributes to psychological strain, particularly among mothers, by disrupting freedom, rest, and personal balance [5]. These findings suggest that individuals with higher well-being levels may approach the transformative demands of parenthood more cautiously. In sum, the desire to have children appears to be shaped by both positive and negative emotional processes. This bidirectional mechanism indicates that fertility motivations cannot be explained through linear models based solely on positive affect; rather, they require a more dynamic and context-sensitive framework. The desire for children may thus be understood as both a life-enriching aspiration and a psychological strategy for coping with distress and maintaining emotional equilibrium.

Another key finding of this study is that egalitarian gender attitudes were associated with a higher desire to have children. This result aligns with complex yet explanatory patterns observed in studies conducted across different cultural contexts. Generally, in Western societies, egalitarian gender attitudes tend to support fertility intentions, whereas in Eastern contexts, traditional gender roles remain more influential. For instance, studies conducted in European settings have shown that partnerships grounded in gender equality are more likely to foster the desire for children [12, 13], and that both egalitarian and traditional men may exhibit higher fertility tendencies [41]. Hudde’s macro-level analyses further revealed that fertility rates increase in societies characterized by a high degree of gender-based consensus [42, 43], whereas Hudde and Engelhardt found that attitudinal discrepancies between partners reduce the likelihood of having a first child [44]. In this regard, egalitarianism emerges not merely as an individual value but also as an indicator of societal cohesion and harmony in role-sharing within intimate relationships.

Data from Western societies indicate that this relationship often follows a U-shaped pattern. Arpino, Esping-Andersen, and Pessin, along with Galdauskaitė, found that fertility tends to be higher in both the most traditional and the most egalitarian contexts, whereas moderate gender attitudes are associated with lower fertility levels [16, 45]. Similarly, Lappegård, Neyer, and Vignoli showed that gender role orientations shape experiences of motherhood, fatherhood, and public participation in different ways, while Hiekel and Begall found that egalitarian individuals who do not place parenthood at the center of life exhibit the lowest fertility intentions [46, 47]. Han, Gowen, and Brinton demonstrated that the “egalitarian familism” model may limit the desire for a second child among working mothers, whereas Bovenberg highlighted that flexible work policies can simultaneously promote both employment and fertility [48, 49]. Camussi et al. further emphasized that the fertility gap often stems from a discrepancy between women’s ideal number of children and the number they perceive as feasible within their economic and professional circumstances [50]. In a related vein, Shang and Yin found that egalitarian individuals in the United States tend to aspire to smaller families, suggesting that the level of cultural and economic prosperity may influence the direction of this association [51].

In contrast, patterns in East and Southeast Asian societies appear markedly different. Studies conducted in China [14, 52] have shown that while traditional gender roles tend to increase fertility, egalitarianism may produce opposite effects in certain regions. Lin further noted that egalitarian women exhibit lower fertility intentions, whereas this trend is reversed among single individuals [53]. In Japan, Yu and Kuo found that as the number of children increases, women tend to adopt less traditional gender attitudes [54]. Similarly, in Iran, Golmakani et al. reported that women who endorse gender stereotypes display higher fertility [15]. Consistent patterns have emerged elsewhere in Asia and Oceania. In South Korea, Tan demonstrated that despite women’s ideal of having two children, inconsistencies between marital and parental attitudes contribute to low fertility rates [55]. In Kazakhstan, Kan found that equal sharing of domestic labor may actually reduce fertility intentions [56], whereas in Australia, Holton, Fisher, and Rowe showed that traditional motherhood attitudes are associated with a greater desire for larger families [57].

These contrasting findings indicate that the relationship between gender roles and fertility cannot be interpreted independently of its cultural, economic, and political context. In Western societies, egalitarianism is often reinforced through individual empowerment and state-supported parenting policies. In contrast, in Eastern societies—particularly in cultures where patriarchal family structures persist—egalitarian values may weaken fertility motivations. Turkey occupies a transitional position between these two tendencies. On one hand, urbanization, education, and the increasing participation of women in the labor force have strengthened egalitarian family values; on the other, traditional norms that idealize motherhood and caregiving continue to hold strong symbolic significance. Therefore, the finding that egalitarian attitudes in Turkey increase the desire to have children can be explained by their dual function: supporting women’s autonomy and quest for meaning while partially aligning with the cultural ideology of motherhood. In summary, the link between gender roles and fertility is shaped by the interaction of couple-level harmony, social consensus, and cultural norms. The Turkish case represents a dynamic transitional sphere between the egalitarian model of the West and the traditional patterns of the East. This finding highlights that the desire to have children is not merely a function of individual happiness or economic opportunity, but is deeply embedded within social value systems, perceptions of gender equality, and ongoing processes of cultural transformation.

According to the findings, no significant relationship was found between perceived social support and the desire to have children. The literature often frames social support as a factor that enhances fertility by sharing parental burdens, reducing costs, and increasing emotional security [58, 59]. The lack of consistency with this expectation suggests that social support is not a unidirectional determinant but a context-sensitive construct with indirect functions. Indeed, a study conducted in Iran initially found social support to be significant, but its effect disappeared in multivariate analyses once demographic factors were introduced [10]. Similarly, European studies have shown that support mechanisms become more salient in intentions for second and third births, yet their effects are most visible when institutional supports are weak [9, 60]. Moreover, the impact of support may vary depending on how it is perceived by individuals. Clarke et al. demonstrated that support is linked not only to fertility but also to subjective well-being and autonomy [61], while Clarke, Taket, and Graham emphasized that it operates through processes of expectation, mobilization, and meaning-making [62]. In the same vein, Graham, McKenzie, Haintz, and Dennis found that the role of support differs according to the type of decision, social network, and lived experience—indicating that it does not necessarily translate into fertility intentions [11].

The structure of social networks also plays a determining role. Bernardi and Klärner, Rossier and Bernardi, and Keim-Klärner et al. argued that fertility decisions are shaped through social learning, pressure, normative guidance, and practical assistance, noting that support facilitates the *implementation* of fertility intentions rather than directly determining them [63–65]. This function also varies across life stages. For example, Pink found that maternal caregiving support accelerates first-birth decisions [66]; Goisis observed that younger mothers receive greater parental support [67]; and Mabetha et al. demonstrated that social support during pregnancy facilitates adjustment to motherhood [68]. Additionally, Zabak et al. showed that the timing of childbearing is influenced by economic conditions and cultural norms, indicating that even when support is available, parenting decisions may still be postponed [69]. In summary, social support is not always a factor that increases fertility; its influence is contingent upon demographic conditions, institutional structures, social networks, and life-stage contexts. The present findings reflect this multidimensionality, suggesting that support does not directly explain the *intention* to have children but rather mediates the processes through which such intentions are formed, delayed, or enacted.

In addition to the findings of the main model, group-based analyses indicated that women’s desire to have children varies significantly across different sociodemographic characteristics. The increase in the desire to have children with higher levels of education may be associated with greater access to resources, enhanced capacity for future planning, and higher perceived readiness for parenthood. This finding is consistent with prior research emphasizing the role of education, labor force participation, and life-course planning processes in shaping the timing and realization of fertility behaviors [1].

Findings related to parental status and number of children further revealed that individuals without children and those with fewer children reported a higher desire to have children, whereas this desire declined as the number of children increased. This pattern suggests that fertility behavior is dynamically shaped by individuals’ existing parental experiences and current number of children, indicating that the relationship between desired and actual fertility is not static [70]. Similarly, the observed decrease in the desire to have children with increasing marital duration may be interpreted within a life-course perspective, reflecting shifting priorities and the increasingly delayed and complex nature of transitions to adulthood [71].

Regarding employment status, the finding that employed women reported a higher desire to have children cannot be explained solely by economic security. Rather, factors such as perceived job control, autonomy, and work–family balance appear to play a crucial role in shaping fertility intentions. Indeed, higher levels of perceived control over work have been associated with stronger fertility intentions, whereas work–family conflict and job strain may weaken such intentions [72]. In addition, broader socioeconomic conditions and partnership contexts also influence childbearing decisions, highlighting the importance of employment quality and social context in the formation of fertility intentions [73].

Furthermore, the finding that individuals using contraceptive methods reported higher levels of desire to have children suggests that such desire is not indicative of unplanned behavior, but rather reflects intentional and temporally regulated fertility intentions. This interpretation aligns with theoretical frameworks positing that fertility behavior unfolds through a multi-stage process involving motivations, desires, and intentions, with intentions representing the most proximal determinant of behavior [74]. Moreover, the higher desire to have children among individuals without chronic illness underscores the role of physical health as an enabling factor in reproductive decision-making and future planning [73]. Taken together, these findings indicate that the desire to have children cannot be explained by single factors; rather, it emerges from the interplay of individual characteristics, relational dynamics, and broader sociocultural contexts. This multidimensional structure is consistent with the literature emphasizing that fertility behavior is shaped by determinants operating at micro, meso, and macro levels [6].

### Limitations and strengths

While this study provides valuable insights through its large and multi-centered sample (*N* = 1,582), several limitations should be noted. First, as the snowball sampling method is non-probabilistic, the representativeness of the sample is limited. Second, the cross-sectional design does not allow for causal inferences. Third, the use of self-report measures carries a potential risk of social desirability bias. Additionally, a substantial proportion of participants were concentrated in the Marmara Region, resulting in a partial imbalance in regional distribution. This concentration also reflects Türkiye’s demographic reality, as the Marmara Region is the country’s most densely populated and socioeconomically dynamic area. Thus, while the uneven distribution poses a potential limitation, it simultaneously provides insight into the central tendencies of social diversity. Finally, since the study sample consisted solely of married women, the findings cannot be generalized to individuals with different marital statuses or sociocultural backgrounds. Despite these limitations, the study has notable strengths, including its large participant pool, data collection across all seven geographical regions of Türkiye, and its comprehensive approach that evaluates the individual impacts of psychological, social, and societal variables.

## Conclusion and implications

This study demonstrates that the desire to have children extends far beyond individual psychological processes; it is a multidimensional phenomenon shaped at the intersection of marital satisfaction, emotional experience, gender role attitudes, and social support mechanisms. The findings reveal that decreased marital satisfaction and heightened negative emotions can trigger a stronger desire for children, while egalitarian gender attitudes strengthen this desire. In contrast, social support functions not as a direct determinant but as a context-dependent and indirect factor. This pattern underscores that the desire to have children cannot be explained solely by happiness or rational decision-making; rather, it represents a multilayered process intertwined with meaning-making, relational compensation, identity construction, and societal values.

The unique contribution of this research lies in its integrative approach: examining women’s fertility intentions across four interconnected levels—individual (emotional state), relational (marital satisfaction), social (perceived support), and cultural (gender role attitudes). Testing these variables together with a large, multi-center sample provides both a context-specific understanding of Turkey and a fresh perspective on global fertility debates. Particularly, the inverse relationships found between positive and negative emotions offer novel insight, revealing that parenting motivation is not solely a reflection of positive life circumstances but may also emerge as a coping mechanism for negative affect and as part of an existential search for meaning.

In light of these findings, future research should employ longitudinal and cross-cultural comparative designs to further explore these dynamics across diverse life stages. Theoretically, fertility motivation should be conceptualized through the multidimensional interactions between individual, relational, and societal levels. From a policy perspective, developing measures that facilitate work–family balance, strengthen gender equality, and enhance the accessibility of social support—not only in economic but also emotional and normative dimensions—will contribute to shaping childbearing intentions under healthier and more equitable conditions.

## Data Availability

The datasets generated and analyzed during the current study are available from the corresponding author on reasonable request.
